# LcDel: deletion variation detection based on clustering and long reads

**DOI:** 10.3389/fgene.2024.1404415

**Published:** 2024-05-10

**Authors:** Yanan Yu, Runtian Gao, Junwei Luo

**Affiliations:** School of Software, Henan Polytechnic University, Jiaozuo, China

**Keywords:** deletion, structural variation, long read, clustering, hierarchical-clustering

## Abstract

**Motivation:** Genomic structural variation refers to chromosomal level variations such as genome rearrangement or insertion/deletion, which typically involve larger DNA fragments compared to single nucleotide variations. Deletion is a common type of structural variants in the genome, which may lead to mangy diseases, so the detection of deletions can help to gain insights into the pathogenesis of diseases and provide accurate information for disease diagnosis, treatment, and prevention. Many tools exist for deletion variant detection, but they are still inadequate in some aspects, and most of them ignore the presence of chimeric variants in clustering, resulting in less precise clustering results.

**Results:** In this paper, we present LcDel, which can detect deletion variation based on clustering and long reads. LcDel first finds the candidate deletion sites and then performs the first clustering step using two clustering methods (sliding window-based and coverage-based, respectively) based on the length of the deletion. After that, LcDel immediately uses the second clustering by hierarchical clustering to determine the location and length of the deletion. LcDel is benchmarked against some other structural variation detection tools on multiple datasets, and the results show that LcDel has better detection performance for deletion. The source code is available in https://github.com/cyq1314woaini/LcDel.

## 1 Introduction

**FIGURE 1:**
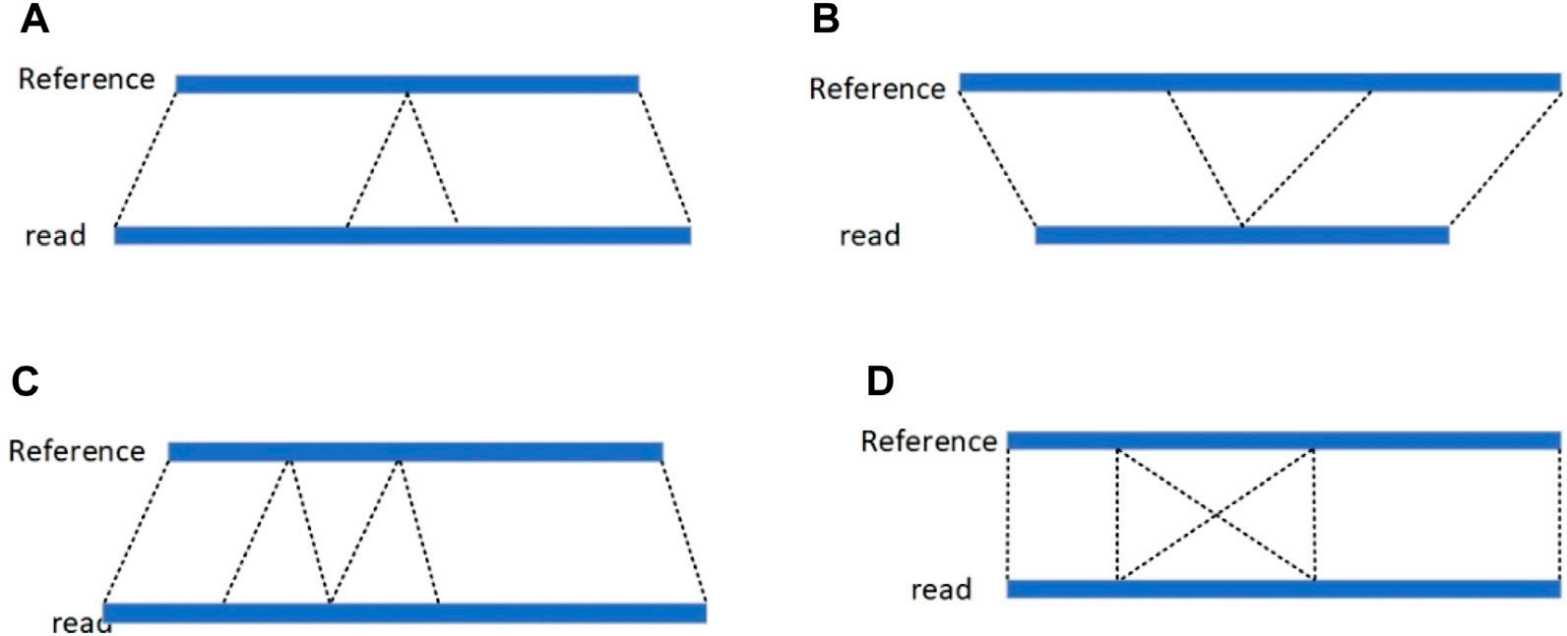
**(A)** insertion; **(B)** deletion; **(C)** duplication; **(D)** inversion (deletion is the loss of a portion of a chromosome; an insertion is the insertion of a portion of a chromosome; a duplication is the repetition of a portion of a chromosome; and an inversion is the reverse complementation operation of a portion of a chromosome.).

Genome sequences are very different between species, even within the same species. Genome variation refers to heritable changes in the composition or arrangement of base pairs at the molecular level of a gene, including single nucleotide variants, indels, structural variants (He et al., 2009), and copy number variants. A single nucleotide variation refers to the variation of one nucleotide base to another under the influence of certain factors; indels refers to the addition or subtraction of a small fragment to the genome that occurs within 50 bp of the length of the small fragment; Copy Number Variation refers to a rearrangement of the genome that has occurred and generally refers to an increase or decrease in the copy number of a genomic segment that is 1 kb or more in length; Structural Variations refer to mutations that occur on chromosomes in segments larger than 50 bp, including forms such as insertions, deletions, duplications, and inversions (Figure 1 below). Deletions account for a certain proportion of structural variants and have a large impact on the human body. Deletions in some genomes may lead to disease (Beyter et al., 2021), for example, deletions of genes related to the nervous system may lead to Huntington’s chorea, and deletions of key genes may lead to cystic fibrosis and autism (Aganezov et al., 2020). Therefore, the detection of deletion variants can provide more precise information for the diagnosis, treatment and prevention of diseases.

Genome sequencing technology has a significant impact on the detection of structural variants, and sequencing technology has gone through the first generation of sequencing technology, the second generation of sequencing technology, and the third generation of sequencing technology. The first-generation sequencing technology is known as Sanger sequencing technology (Sanger et al., 1977) and is widely used for genome sequencing. The read length of the first-generation sequencing technology can reach 1,000 bp with an accuracy of 99.99%, but the shortcomings of high sequencing cost and low throughput restrict its further application. Second-generation sequencing technology is also known as high-throughput sequencing (Maxam and Gilbert, 1992), which has the advantages of low cost, low sequencing error rate and high throughput, but the sequencing reads are shorter in length, which is more suitable for the detection of shorter structural variations, and the detection of structural variations in repetitive regions and regions with a high GC content has some difficulties. Third-generation sequencing technology, also known as single-molecule real-time technology (Korlach et al., 2010), is capable of directly sequencing longer DNA fragments and providing more comprehensive genomic information, but the sequencing error rate is high and therefore structural variants are not detected in sufficiently accurate locations. Cycle-consistent sequencing technology (Wenger et al., 2019) can sequence highly accurate long reads that cover repetitive and GC-rich regions of the genome, and can therefore be well suited for detecting structural variants.

Hi-C sequencing (de Wit and de Laat, 2012) is a high-throughput sequencing technology used to study the three-dimensional structure of chromosomes and genome interactions, which joins DNA fragments from different chromosomal regions by enzymatic cleavage and ligation techniques to form a DNA molecular library, which is then subjected to high-throughput sequencing to obtain sequences of multiple DNA reads. A number of methods for structural variation detection based on Hi-C reads have emerged, such as HiNT (Wang et al., 2020), HiCNV (Chakraborty and Ay, 2018), HiSV (Li et al., 2023), EagleC (Wang et al., 2022), etc. HiNT is a method for detecting interchromosomal translocations using Hi-C read. It utilizes a 1 Mb bin chromosome contact matrix as input. HiNT first calculates the Gini coefficient and maximum contact frequency of the interchromosomal contact matrix to identify potential translocated chromosome pairs. Then, it employs the breakpoint function from the R package ‘struchanger’ to approximate the breakpoints of the translocation. Finally, it utilizes an algorithm based on soft-clipped read counts to achieve precise breakpoint detection at single base pair resolution. HiCNV is a method for detecting copy number variations (CNVs) based on Hi-C read. It first processes the contact counts at the level of individual restriction enzyme fragments to utilize Hi-C data with as high resolution as possible. HiCNV calculates one-dimensional read coverage for each restriction enzyme, normalizes for GC content, mappability, and fragment length, smoothes using kernel density estimation, and finally identifies potential CNV segments using a hidden Markov model. HiSV is a structural variation detection method based on a significance detection model, capable of identifying large-scale structural variations from Hi-C read. Firstly, HiSV calculates a distance-normalized Hi-C contact matrix to avoid interference from strong interactions on the diagonal. Then, HiSV computes the local spatially weighted dissimilarity for each pixel to measure significance, thus separating significant regions from complex backgrounds. Finally, HiSV uses a global variation segmentation approach to partition sparse significant subsets into segments, considering a segment as a structural variation event if the interaction frequency after segmentation exceeds a predefined threshold. EagleC transforms the problem of identifying structural variations from Hi-C maps into a multi-label image classification problem. It is an ensemble learning framework based on 50 different models and utilizes convolutional neural networks as individual models for prediction. Additionally, EagleC proposes a data augmentation algorithm to ensure a balanced distribution of samples across different types of structural variations and genomic regions. These methods compare the interaction levels between normal and variant regions; large variant regions exhibit clear interaction patterns, while small variant regions exhibit less distinct interaction patterns. Therefore, these methods perform well in detecting large variant regions but less effectively for small ones. Moreover, since cancer cell lines often contain a higher proportion of large variant regions, these methods are effective in identifying variant regions in cancer cell lines and can be used for disease prediction.

The recently emerged Pore-C technology (Zhong et al., 2023), on the other hand, refers to a new technology that combines chromatin conformation capture technology with Nanopore sequencing technology to capture the information of chromatin multiregional interactions, and is able to form a single long read from multiple sequence fragments that are in close proximity to each other in three-dimensional space. This technique is capable of generating long reads on a genome-wide scale; however, these techniques generate less information about interactions in regions larger than three. Because of the complexity and diversity of structural variants, methods for detecting structural variants based on Pore-C reads are not yet available.

Most of the deletion variant detection tools (Mahmoud et al., 2019) that currently exist are based on short or long read, and although short read have a low sequencing error rate, they are short in length and do not completely find the deletion site. Although the sequencing error rate of long reads is high, the length is relatively long and it can span the deletion breakpoints well, so most of the tools nowadays detect the deletion by long reads, mainly by utilizing two methods, which are traditional method or deep learning.

The traditional method mainly involves first extracting candidate loci by characterizing various variants, and then clustering the candidate loci to determine the exact variant loci and length. CuteSV (Jiang et al., 2022) analyzes the characteristics of each type of structural variation and uses them to find potential loci for each variation separately, and clusters and further refines the clustering of read from heterozygous ratios in localized regions to accurately distinguish between pure and heterozygous variants. Finally, a few specific rules are used for structural variant detection and genotyping. Svim (Heller and Vingron, 2019) also collects structural variant features from the alignment files of the input sequences, then clusters the detected features using a clustering method based on graph and structural variant feature distance metrics, and finally outputs the final result by merging multiple structural variant events. Sniffles (Sedlazeck et al., 2018) uses the results from the NGMLR alignment as input and utilizes features from the segmented reads alignment, high mismatch regions, and coverage to identify structural variants. To overcome the high error rate in the reads, sniffles also evaluates candidate structural variants based on features such as length, location, and consistency of breakpoints. SKSV(Liu et al., 2021) is a skeleton-based structural variation detection analysis toolkit that performs pseudo alignment from reads and generates a alignment skeleton through sparse dynamic programming. The generated alignment skeleton supports rapid read finding and non-collinear segments in the alignment skeleton indicate potential structural variant events. Compared to other methods, SKSV is extremely fast and achieves high sensitivity and accuracy in both structural variant detection and genotyping. Svsearcher (Zheng et al., 2023) differs from previous methods in that it first finds candidate structural variant regions by variant characterization, then clusters read within the candidate regions to find candidate structural variants, and sets a stricter criterion to filter out erroneous structural variants.

Structural variation detection based on deep learning is mainly based on first extracting various features according to the type of structural variation, and then through the continuous training of the neural network, and then through the neural network to make predictions. INSnet (Gao et al., 2023) is a deep learning-based insertion variant detection method that firstly divides the reference genome into contiguous sub-regions and acquires five features for each locus. INSnet uses a convolutional neural network to extract variant features and a gated recurrent unit to analyze connections between subregions. MAMnet (Ding and Luo, 2022), a structural variation detection method based on the combination of convolutional neural networks and long and short-term memory networks, achieved a better F1-score compared to other comparison tools. SVcnn is a deep learning method that can accurately detect deletion, insertion, duplication and inversion variants. SVcnn (Zheng and Shang, 2023) first identifies candidate structural variant regions from the BAM file, then converts the candidate structural variant regions into images and constructs a LetNet model, which filters out the erroneous structural variants and outputs the final structural variants. cnnLSV (Ma et al., 2023) is also a deep learning based structural variation detection method that utilizes alignment information of long reads and convolutional neural networks to achieve overall higher performance and utilizes principal component analysis and k-means clustering algorithms to efficiently eliminate mislabeled samples during the training model phase. The results show that cnnLSV outperforms existing methods in detecting insertions, deletions, inversions and duplicate variants.

The traditional method ignores the occurrence of two different lengths of deletion variants at the same locus and directly clusters the candidate deletion sites, which may affect the final results of the detection of deletion variants. And deep learning can take a lot of time when extracting features and training. Therefore, this paper proposes an effective deletion variant detection algorithm LcDel. LcDel firstly merges the deletion variants that are closer when finding candidate deletion sites, then uses two clustering algorithms to perform the first level of clustering according to the length of deletion and uses hierarchical clustering to perform the second level of clustering, respectively, and finally filters out the candidate clusters that do not match to identify the location and length of deletion.

## 2 Methods

**FIGURE 2:**
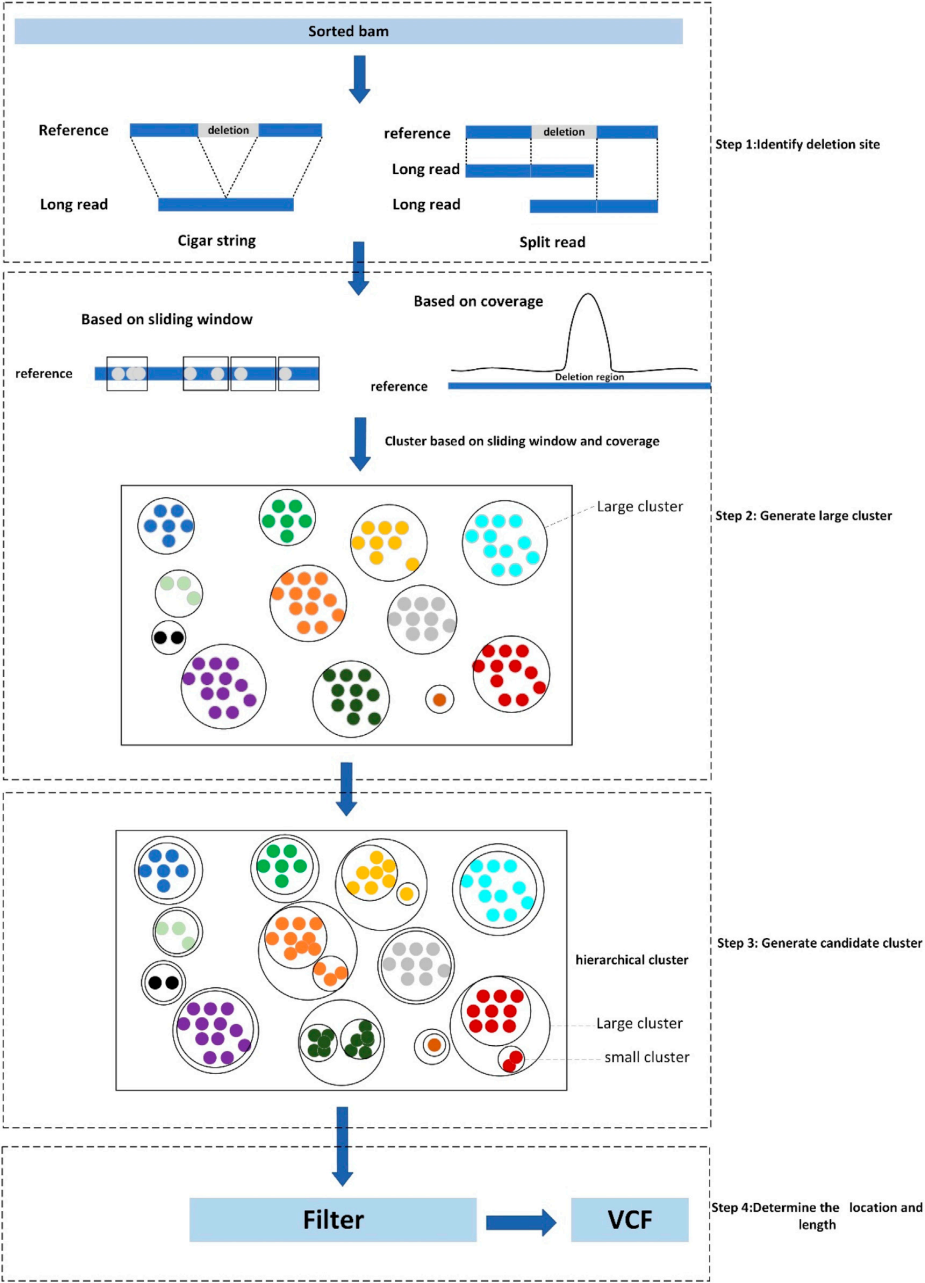
Workflow diagram of LcDel, step1 denotes identification of deletion sites, step2 denotes the first level of clustering to generate large clusters, step3 denotes the second level of clustering to generate candidate clusters, and step4 denotes the determination of the location and length of the deletion.

LcDel is a long reads-based deletion variant detection method where the input is a sorted bam file including the alignments between long reads and genome reference. There are four main steps in LcDel: 1) Identification of candidate deletion sites by intra-read alignment and inter-read alignment; 2) Multiple large clusters are generated by performing the first layer of clustering based on deletion lengths using sliding window-based and coverage-based methods, respectively; 3) Generate candidate clusters based on the differences between deletion lengths for large clusters using hierarchical clustering; 4) Set the support read threshold to filter out non-compliant candidate clusters and determine the location and length of the deletion. LcDel workflow is shown in Figure 2 below.

### 2.1 Identify candidate deletion site

Since small deletion variants are aligned to the reference genome, the alignment tool will directly display the deletion information in the cigar string, while large deletions are not directly displayed in the cigar string, but will be aligned to two non-contiguous regions of the same chromosome by splitting the reads, LcDel identifies candidate deletion loci by intra-read alignment and inter-read alignment, respectively.

LcDel first filters out alignments with mapping quality scores lower than 20 and unaligned ones, and then finds the ‘D’ identifiers with lengths greater than 30 in the cigar string, records the position and length of the deletion event on the reference genome, considers it as a deletion site and represents it as a quaternion Dt=(chr, start, svlen, end), where chr denotes the chromosome that the reads are aligned, start, and end denote the start and end positions of the deletion on the chromosome, respectively, and svlen denotes the length of the deletion on the chromosome. Due to the high sequencing error rate of long reads, which may result in a single deletion region being split into multiple smaller deletion regions during sequencing and alignment, it is necessary to determine whether merging can be performed if there are two deletion sites on the same read. For two quaternions Dt_1_= (chr_1_, start_1_, svlen_1_, end_1_) and Dt_2_= (chr_2_, start_2_, svlen_2_, end_2_) for the same read, where Dt_1_ is assumed to be located in front of Dt_2_, calculate the gap by using gap = start_2_-end_1_, and if 0<gap≤ 30, then Dt_1_ and Dt_2_ are combined into a quaternion Dt= (chr_1_, start_1_, svlen_1_+svlen_2_, end_2_), the new quaternion represents a large deletion variant.

**FIGURE 3:**
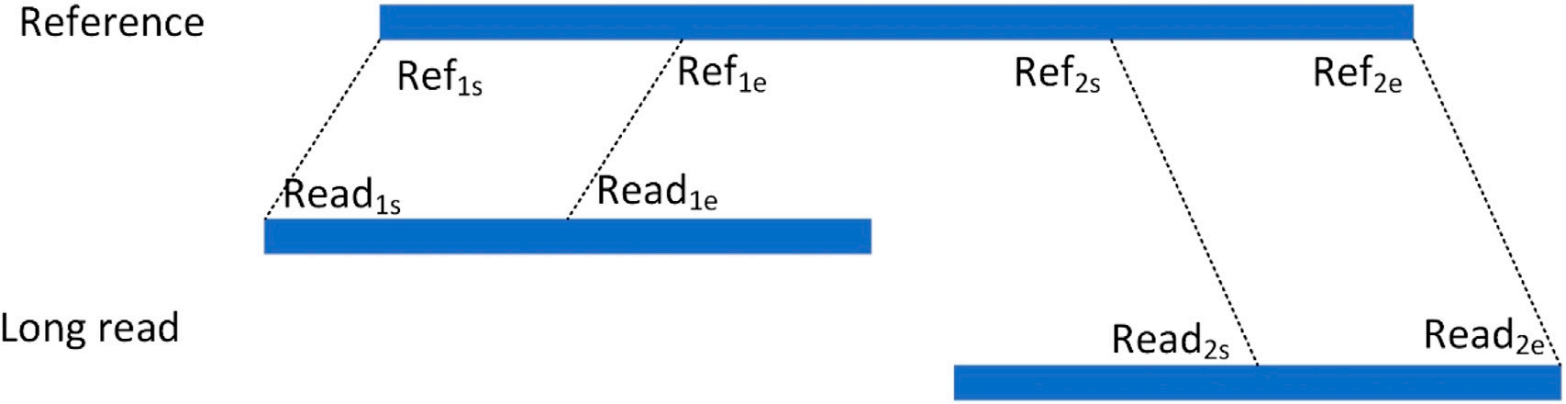
Split alignment of long read.

For an alignment containing segmented reads, each matched read is represented as a hexadecimal Sig=(chr, Ref_s_, Ref_e_, Read_s_, Read_e_, orient), respectively, where chr denotes the chromosome to which the read is aligned, Ref_s_ and Ref_e_ denote the start and end points of the read alignment to the reference genome, respectively, Read_s_ and Read_e_ denote the start and end points of the segment of reads that are matched to the reference genome in the reads, respectively, and orient denotes the direction in which the read is aligned to the reference genome. As shown in Figure 3, the read is aligned to the reference genome due to the presence of variants resulting in splitting the read into two segments to be aligned to the reference genome separately, denoting the two alignments as the hexameric group Sig_1_= (chr_1_, Ref_1s_, Ref_1e_, Read_1s_, Read_1e_, orient_1_) and Sig_2_= (chr_2_, Ref_2s_, Ref_2e_, Read_2s_, Read_2e_, orient_2_), respectively. For two read segments of a split read comparison, which are aligned to the same chromosome in the same direction, i.e., chr_1_ = chr_2_ and orient_1_ = orient_2_, the spacing Distance_ref on the chromosome, the spacing Distance_read on the read, and the difference in their spacing Distance are computed, respectively. The setting of the upper limit of the distance threshold is described in detail in the results section 3.5.

$$\left\{\begin{array}{l} Distance\_ref={Ref}_{2s}-{Ref}_{1e}\\ Distance\_read={Read}_{2s}-{Read}_{1e}\\ Distance=Distance\_ref-Distance\_read \end{array}\right.$$

If Distance lies between the interval [50, 100,000], it indicates that this splitting read contains a deletion event, which is considered as a candidate deletion site and represented as a quaternion Deletion= (chr, Ref_1e_, Distance, Ref_2s_).

### 2.2 Generate large cluster

Clustering is commonly used for grouping data points in a dataset with similar characteristics into one category to help us better understand and utilize the information in the dataset, discover patterns and regularities in the data, and provide useful tasks for subsequent prediction and classification. The traditional methods for structural variant detection are generally to first find potential candidate variant sites through coverage, split reads and other features, and then filter the clusters with higher confidence as candidate clusters through clustering, and find the appropriate variant sites from the candidate clusters as the final result. In structural variation detection, a sliding window-based clustering method is usually used, which can effectively cluster candidate loci representing the same variant site together to facilitate structural variation detection. Clustering methods based on sliding windows need to set the window size in advance, and since the window size is fixed, when the window is set too large or too small it will result in the final finding of structural variants that are not complete. Since the coverage of the deletion region is lower than that of the normal region, the deletion region can be found by observing the coverage, so LcDel clusters in the first layer of clustering according to the length of deletion variation based on the two clustering methods of the sliding window and coverage, respectively, which can effectively cluster the deletion events that are mutated at the same locus together.

LcDel first sets a length threshold of 2000 and then refers to deletion lengths less than this threshold as small candidate deletion sites and deletion lengths greater than this threshold as large candidate deletion sites. The small candidate deletion sites are then clustered using a coverage-based clustering method, while the large candidate deletion sites are clustered using a sliding window-based clustering method.

**FIGURE 4:**
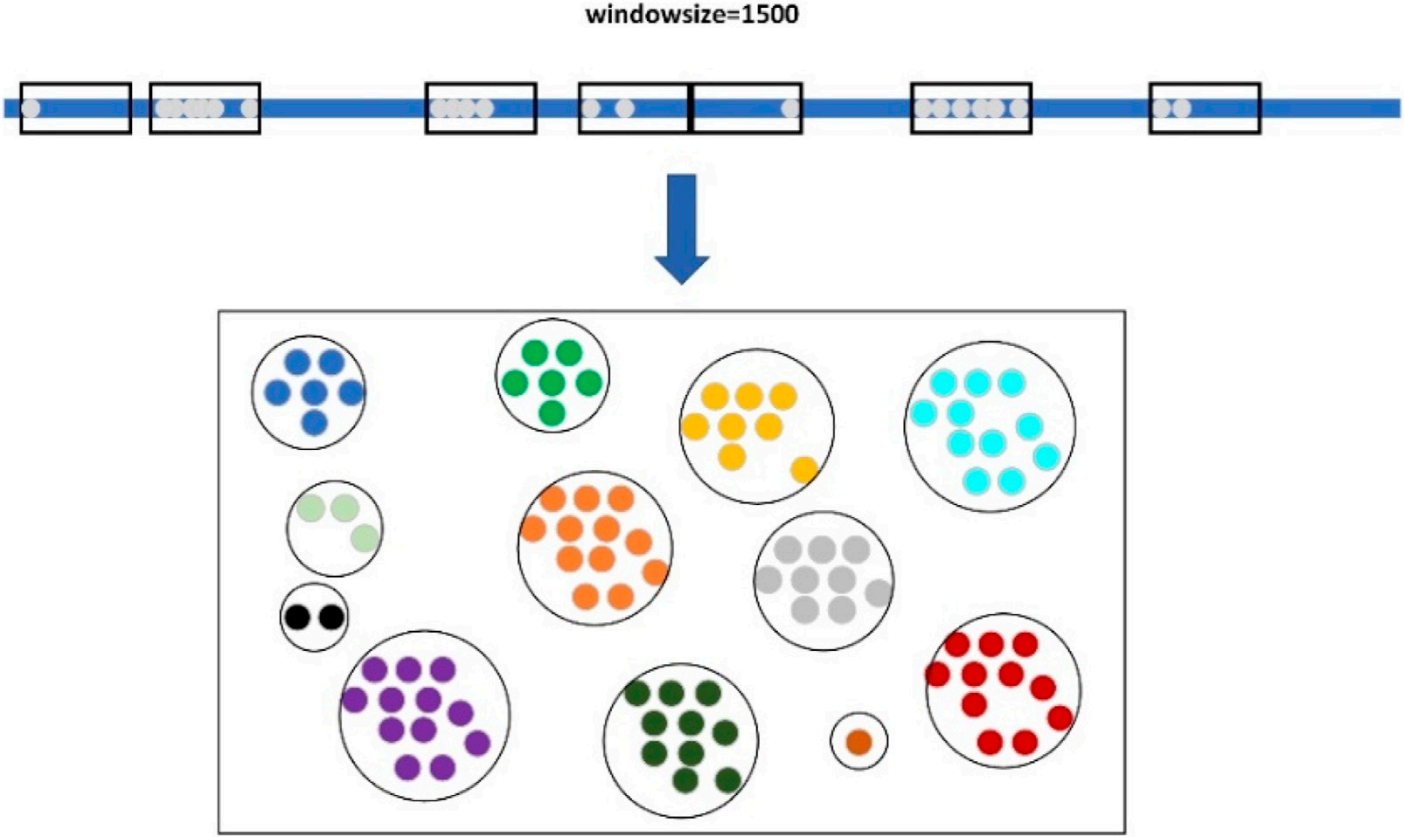
Clustering of large candidate deletion sites based on sliding windows.

When clustering large candidate deletion sites, LcDel first sorts the large candidate deletion sites in ascending order according to their position on the reference genome, and then sets up a window of length 1,500, as shown in Figure 4 below. The starting position of the sliding window is the position of the first large candidate deletion site on the reference genome, and then keep sliding the window, if there is no large candidate deletion site in the sliding window at a certain moment, the next large candidate deletion site will be taken as the new starting point of the sliding window directly, and if the sliding window contains a large candidate deletion site, the next sliding window will need to take the end position of the window as the new starting point. In the process of continuously sliding the window, the large candidate deletion sites contained within the sliding window are clustered together to form a cluster, which is considered as a large cluster.

Since the coverage of the deletion region is significantly lower than that of the normal region, analyzing the feature of coverage can detect the deletion region, so the small candidate deletion sites are clustered using the coverage-based clustering method. LcDel sets up a list of chromosomes in the reference genome of length corresponding to the length of the corresponding chromosome, respectively, and the initial values of the list are all 0. Each position in the list corresponds to the corresponding base site on the reference genome. Then LcDel traverses each small candidate deletion site, looks at the region where each small candidate deletion site is located, takes out the list corresponding to the reference genome where the small candidate deletion site is located, and then adds 1 to the value of the list corresponding to the deletion region, and continually continues this process until all the small candidate deletion sites have been traversed. LcDel uses each locus of the reference genome as a horizontal coordinate and the list value corresponding to that locus as a vertical coordinate to build a planar graph.

As shown in Figure 1, step 2, the deletion region will form a shape similar to a mountain peak. Find the interval corresponding to that peak and cluster the small candidate deletion sites within that interval together to form a large cluster. In the first layer of clustering process, based on the sliding window and coverage clustering is carried out for different candidate deletion sites, the two clustering methods are independent of each other, so the two clustering methods are carried out at the same time, and the clustering for each chromosome is also processed in parallel by multi-threading, which makes LcDel extremely fast in the first layer of clustering. At the end of the first layer of clustering, all candidate deletion sites are clustered into multiple large clusters.

### 2.3 Generate candidate cluster

Since one human chromosome is composed of two homologous chromosomes, however, deletion variants of different lengths may have occurred on the two homologous chromosomes, such as deletion events of lengths 108 and 216 at locus 1,120,034 on chromosome 1, respectively. If the large clusters from the first level of clustering are used directly as final candidate clusters, the deletion length at that location may be determined incorrectly when determining the deletion length, affecting the final detection results. In order to separate these deletion variations, LcDel uses a hierarchical clustering method for a second clustering, which makes the clustering results more accurate and helps to determine the subsequent deletion length.

LcDel treats each candidate deletion site in a large cluster as a small cluster, and the average of the lengths of all the deletions in the small cluster is considered as the deletion length of the small cluster. LcDel calculates the length difference between any two small clusters contained in the large cluster each time, and then LcDel merges the two small clusters with the smallest length difference, and keeps iterating this merging process until the final large cluster contains only two small clusters. For two small clusters with deletion lengths of len_1_ and len_2_, respectively, the length difference rate between them can be calculated by the following formula. At this point, candidate deletion sites that support different deletion length variants can be clustered together to form small clusters. Finally, it is necessary to determine whether two small clusters represent the same deletion variant based on the difference in length between them, and to determine whether two small clusters in a large cluster can be merged into a single cluster. If the difference in the length of two clusters is less than 20%, the two clusters are merged into one candidate cluster, otherwise both clusters are considered as candidate clusters.

$$rate=\frac{abs\left({len}_{1},{len}_{2}\right)}{\max\;\left({len}_{1},{len}_{2}\right)}$$

### 2.4 Determine location and length of deletion

Candidate clusters have been identified through the previous two layers of clustering. LcDel then sets a support read threshold that filters out the following two types of candidate clusters: 1) The large cluster contains only one candidate cluster and the number of candidate deletion sites in the candidate cluster is less than the supported read threshold; 2) The large cluster contains two candidate clusters, and the number of candidate deletion sites in the candidate clusters is less than half of the threshold of supported reads. To better illustrate the benefits of splitting our filtering of candidate clusters into two cases, we also benchmarked the HG002 CLR dataset in one case (filtering out candidate clusters smaller than the threshold of supported reads), and the results are shown in Table 1 below. By analyzing LcDel on CLR datasets with different coverage, it can be seen that LcDel can effectively improve the detection of deletion variants when filtered in two cases.

**TABLE 1:** Detection performance of LcDel in different situations.

Coverage		one_situation	two_situation
69X	precision	0.9535	0.9611
	recall	0.9725	0.9832
	F1	0.962	0.9721
35X	precision	0.9387	0.9485
	recall	0.9561	0.9764
	F1	0.9473	0.9623
20X	precision	0.9293	0.9369
	recall	0.9341	0.9446
	F1	0.9316	0.9407
10X	precision	0.9186	0.9273
	recall	0.8563	0.8671
	F1	0.8861	0.8962
5X	precision	0.9412	0.9573
	recall	0.6765	0.6866
	F1	0.7871	0.7997

For the candidate clusters that are left behind, the average of the deletion positions and lengths in that candidate cluster is calculated, and the candidate deletion site with the deletion position and length closest to the average in that candidate cluster is taken as the final result.

## 3 Results

In order to objectively evaluate the detection performance of LcDel for deletion variants, this paper compares LcDel with four of the more frequently used current structural variant detection tools. The four structural variant detection tools, all of which perform variant detection based on long reads, are cuteSV, sniffles, svim, and pbsv. High-confidence deletion variant regions collected by the Genome in a Bottle program were used as the reference standard dataset for this experiment, and Truvari was used to evaluate and record precision, recall, and F1-scores for all experimental results. In order to fully evaluate the deletion detection performance of LcDel, three human sample datasets that are currently more commonly used were selected: HG002 CLR (average length: 7938bp), HG002 CCS (average length: 13,478bp), and detailed information of the datasets is shown in Table 2 below. Additionally, this paper sets appropriate support read thresholds for each detection tool separately, with specific settings for detection performance on each dataset presented in the Detection Performance section.

**TABLE 2:** Description of the dataset.

	HG002 CLR	HG002 CCS
Read Count	2,915,733	6,596,012
Average Length	7,938	13,478
Coverage	69X	28X

In the following experiments, we detected deletion variation on chromosomes 1–22. The structural variation detection methods based on deep learning commonly selects a portion of chromosomes as the training set and a portion of chromosomes as the validation set. These models are continuously trained through the training and validation sets, and finally the remaining chromosomes are predicted in the test set. Therefore, it is not appropriate to compare LcDel with the methods using deep learning.

### 3.1 Detection performance of the structural variation detection tools on the CLR dataset

First, we benchmark LcDel, cuteSV, svim, sniffles and pbsv detection tools on the HG002 CLR dataset, and the experimental results are shown in Table 3 below. In order to fully evaluate the deletion detection performance of LcDel on datasets with different coverage, we also randomly downsampled the HG002 CLR dataset to 35X, 20X, 10X and 5X and benchmarked it. For datasets with 69X, 35X, 20X, 10X and 5X coverage, the support read thresholds were set to 10, 5, 3, 2 and 2, respectively.

**TABLE 3:** Performance comparison of SV detection tools on CLR Dataset.

Coverage		LcDel	cuteSV	Sniffles	Svim	pbsv
69X	Precision	0.9611	0.9557	0.964	0.9595	0.9617
	Recall	0.9832	0.9436	0.9438	0.9461	0.9472
	F1	0.9721	0.9496	0.9538	0.9527	0.9544
35X	Precision	0.9485	0.9527	0.9641	0.957	0.9634
	Recall	0.9764	0.9361	0.9261	0.9368	0.9368
	F1	0.9623	0.9443	0.9447	0.9468	0.9499
20X	Precision	0.9369	0.9504	0.9622	0.958	0.9609
	Recall	0.9446	0.9091	0.8794	0.9052	0.8736
	F1	0.9407	0.9293	0.919	0.9309	0.9152
10X	Precision	0.9273	0.9434	0.9559	0.9396	0.967
	Recall	0.8671	0.8377	0.7918	0.8389	0.6496
	F1	0.8962	0.8874	0.8662	0.8864	0.7772
5X	Precision	0.9573	0.9656	0.9649	0.9647	0.973
	Recall	0.6866	0.6632	0.6285	0.6586	0.4042
	F1	0.7997	0.7864	0.7612	0.7828	0.5712

Compared to the other four popular structural variation detection tools, as shown in Table 3, LcDel achieves the highest recall and F1-score on all coverage CLR datasets. For the 69X dataset, the precision of LcDel detection is not the highest, but it is only 0.3% less than the first place, while the recall and F1-score are the highest, with the recall being 3.6% higher than the second place and the F1-score being 1.77% higher than the second place, which indicates that LcDel has a better performance of deletion detection on the high coverage dataset. For the CLR dataset with 35X coverage, LcDel detection had the lowest precision, 1.5% lower than the first place, but the recall was 0.9% higher than the second place and the F1-score was 1.2% higher than the second place. For the 20X CLR dataset, the precision of LcDel detection was also the lowest, 2.5% lower than the first place, but the recall was 3.5% higher than the second place, achieving the highest F1-score. The performance of structural variant detection tools for deletion variant detection decreases with decreasing sequencing depth. For the 10X dataset, the precision of LcDel detection was 3.9% lower than the first place, but the recall was 2.8% higher than the second place, achieving the highest F1-score. For the dataset of 5X, the precision of LcDel detection is 1.5% lower than that of pbsv, but the recall is 28.24% higher than that of pbsv, achieving the highest recall and F1-score, which indicates that LcDel has a better performance of deletion detection on CLR datasets of different coverage.

### 3.2 Performance of structural variation detection tools on different deletion lengths

In order to evaluate the performance of structural variant detection tools for different deletion lengths, in this paper, the variant lengths are categorized into five intervals of [50, 200], [200, 500], [500, 1,000], [1,000, 2000], and [2000+] for benchmarking respectively. The structural variation detection tool was benchmarked on the 69X、10X and 5X datasets of the HG002 CLR and the results are shown in Table 4 and 5 below, respectively.

**TABLE 4:** Comparison of detection performance for different deletion lengths.

Coverage	Interval		LcDel	cuteSV	Sniffles	Svim	pbsv
69X		Precision	0.9394	0.936	0.9514	0.9446	0.947
	50–200	Recall	0.9768	0.9018	0.9208	0.9217	0.9416
		F1	0.9577	0.9186	0.9359	0.933	0.9443
		Precision	0.9846	0.9718	0.9778	0.9754	0.9744
	200–500	Recall	0.9831	0.9831	0.9846	0.9792	0.9669
		F1	0.9838	0.9774	0.9812	0.9774	0.9706
		Precision	0.9692	0.9447	0.9741	0.9489	0.9659
	500–1,000	Recall	0.9594	0.9543	0.9543	0.9441	0.8629
		F1	0.9643	0.9494	0.9641	0.9465	0.9115
		Precision	0.9793	0.9687	0.9639	0.9585	0.9502
	1,000–2000	Recall	0.9844	0.9687	0.9739	0.9635	0.8958
		F1	0.9818	0.9687	0.9689	0.961	0.9222
		Precision	0.9719	0.9386	0.9419	0.9444	0.9354
	2000+	Recall	0.9811	0.9622	0.8679	0.9088	0.9119
		F1	0.9765	0.9503	0.9034	0.9262	0.9235

**TABLE 5:** Comparison of detection performance for different deletion lengths.

	Interval		LcDel	cuteSV	Sniffles	Svim	pbsv
		Precision	0.886	0.9221	0.9383	0.9169	0.9566
	50–200	Recall	0.844	0.7975	0.7568	0.8113	0.6382
		F1	0.8645	0.8553	0.8378	0.8609	0.7656
		Precision	0.9709	0.9626	0.9709	0.9609	0.9768
	200–500	Recall	0.8985	0.8915	0.8754	0.8907	0.7154
		F1	0.9333	0.9257	0.9207	0.9246	0.8259
		Precision	0.95	0.9389	0.9697	0.9389	0.952
10X	500–1,000	Recall	0.868	0.8578	0.8121	0.8579	0.6041
		F1	0.9072	0.8965	0.8839	0.8966	0.7391
		Precision	0.9583	0.9464	0.9491	0.9509	0.9357
	1,000–2000	Recall	0.8385	0.8281	0.776	0.8073	0.5313
		F1	0.8944	0.8833	0.8539	0.8732	0.6778
		Precision	0.9446	0.9283	0.9346	0.9387	0.9302
	2000+	Recall	0.8585	0.8144	0.6289	0.7705	0.5031
		F1	0.8995	0.8677	0.7519	0.8463	0.6531
		Precision	0.886	0.9221	0.9383	0.9169	0.9566
	50–200	Recall	0.844	0.7975	0.7568	0.8113	0.6382
		F1	0.8645	0.8553	0.8378	0.8609	0.7656
		Precision	0.9709	0.9626	0.9709	0.9609	0.9768
	200–500	Recall	0.8985	0.8915	0.8754	0.8907	0.7154
		F1	0.9333	0.9257	0.9207	0.9246	0.8259
		Precision	0.95	0.9389	0.9697	0.9389	0.952
5X	500–1,000	Recall	0.868	0.8578	0.8121	0.8579	0.6041
		F1	0.9072	0.8965	0.8839	0.8966	0.7391
		Precision	0.9583	0.9464	0.9491	0.9509	0.9357
	1,000–2000	Recall	0.8385	0.8281	0.776	0.8073	0.5313
		F1	0.8944	0.8833	0.8539	0.8732	0.6778
		Precision	0.9446	0.9283	0.9346	0.9387	0.9302
	2000+	Recall	0.8585	0.8144	0.6289	0.7705	0.5031
		F1	0.8995	0.8677	0.7519	0.8463	0.6531

The above analysis reveals that LcDel has good detection performance for different deletion variant lengths on both high coverage (69X) and low coverage (10X and 5X) datasets of HG002 CLR.

Compared with the other four commonly used structural variation detection tools, as shown in Table 4, LcDel achieved the highest F1-scores at different deletion lengths, which indicates that LcDel has a better detection effect for different deletion lengths on the 69X dataset of HG002 CLR. On the [50, 200] interval, the precision of LcDel detection was 1.2% lower than the first place, but the recall was 3.5% higher than the second place, and the F1-score was 1.34% higher than the second place, which indicates that LcDel has a better detection effect on small deletion variants. On the [200, 500] interval, although the LcDel detection had the second highest recall, only 0.15% lower than the first place, it achieved the highest precision and F1-score. On the [500,1000] interval, although LcDel did not detect the highest precision, it achieved the highest recall and F1-score. On the [1,000,2000] interval, LcDel achieved the highest precision, recall, and F1-score, which were 1.06%, 1.05%, and 1.29% higher than the second place, respectively. By analyzing in the intervals [200, 500], [500, 1,000] and [1,000, 2000], it was found that LcDel has better performance for large deletion variant detection. On the [2000+] interval, LcDel also achieved the highest precision, recall, and F1-score, which were 2.7%, 1.8%, and 2.6% higher than the second place, respectively, which demonstrated LcDel’s better detection performance even for larger deletion variants.

As shown in Table 5, LcDel achieved the highest recall and F1-score on different intervals, which indicates that LcDel has a better detection effect for different deletion lengths on the 10X dataset of HG002 CLR. On the [50, 200] interval, although the pbsv detection had the highest precision, the LcDel detection had 20.58% higher recall and achieved the highest F1-score. On the [200, 500] interval, LcDel achieved the second highest precision, only 0.59% lower than the first place, but the recall and F1-score were 0.7% and 0.76% higher than the second place, respectively. On the [500, 1,000] interval, although the precision of LcDel detection was 1.97% lower than sniffles, the recall was 5.59% higher, achieving the highest F1-score. LcDel achieved the highest precision, recall, and F1-score on both the [1,000, 2000] and [2000+] intervals.

Although LcDel, cuteSV, sniffles, svim, and pbsv were all detected poorly on the 5X of the HG002 CLR dataset, LcDel still achieved the highest F1 scores on each interval, which suggests that LcDel has a better detection performance for deletion variants of different lengths even at low coverage.

### 3.3 Performance of LcDel on different support read parameters

Too large or too small support reads can affect the performance of detection of deletion variants, in order to evaluate the performance of LcDel in detecting deletion variants under different support reads thresholds, so this paper sets the support read support to 2, 3, 5 and 10 on 69X, 35X and 10X datasets of HG002 CLR to benchmark LcDel, respectively. The test results are shown in Table 6, which shows that on the 69X HG002 CLR dataset, as the number of supported reads continues to increase, the precision continues to increase and the recall continues to decrease, but the F1 score generally increases until a better detection result is achieved at a number of supported reads of 10. On the HG002 CLR dataset of 35X, it was found that LcDel achieves better deletion detection performance when the number of supported reads is set to 5. On the 20X dataset, better deletion detection results are achieved when the number of supported reads is set to 3. From the above analysis, it is found that the larger the support read are set, the greater the precision of LcDel detection and the smaller the recall.

**TABLE 6:** LcDel deletion detection performance at different supported read.

Coverage	Support>=2	Support>=3	Support>=5	Support>=10
69X	0.6094	0.7419	0.8755	0.9611
	0.9965	0.9942	0.9922	0.9832
	0.7563	0.8498	0.9302	0.9721
35X	0.7602	0.8721	0.9488	0.9818
	0.9934	0.9908	0.9764	0.8503
	0.8612	0.9277	0.9624	0.9113
20X	0.8708	0.9393	0.9774	0.9924
	0.9796	0.9444	0.8188	0.4103
	0.922	0.9418	0.8911	0.5806

### 3.4 Detection performance of the structural variation detection tool on the CCS dataset

In order to fully evaluate the performance of LcDel on different datasets for deletion variant detection, in addition to the CLR dataset of HG002, this paper also benchmarked LcDel, cuteSV, sniffles, svim, and pbsv on the CCS dataset of HG002, respectively. In addition to that, in this paper, the CCS dataset is randomly downsampled to 10X and 5X, and the support reads are set to 3, 2, and 1 for benchmarking, respectively, and the results are shown in Table 7. On CCS datasets with different coverage, although none of LcDel’s precision is the highest, its recall is the highest and it achieves a good F1 score, which shows that LcDel can achieve similar deletion detection performance on CCS datasets as other detection tools.

**TABLE 7:** Comparison of the performance of SV detection tools on the CCS dataset of HG002.

Coverage		LcDel	cuteSV	Sniffles	Svim	pbsv
28X	Precision	0.9378	0.9366	0.9487	0.9443	0.9459
	Recall	0.9504	0.9414	0.9399	0.9446	0.9346
	F1	0.9441	0.939	0.9433	0.9444	0.9402
10X	Precision	0.9346	0.9447	0.9525	0.9382	0.9567
	Recall	0.9113	0.9016	0.8787	0.9040	0.8214
	F1	0.9228	0.9226	0.9141	0.9208	0.8839
5X	Precision	0.9109	0.9217	0.9581	0.9164	0.9722
	Recall	0.8818	0.8731	0.6994	0.8743	0.5017
	F1	0.8962	0.8968	0.8085	0.8949	0.6618

### 3.5 Deletion detection performance of LcDel at different distance thresholds

Since reads spanning small deletion regions are compared with the reference genome, the deletion information is displayed directly in the cigar string, e.g., the presence of 100D in the cigar string indicates that the region to which the read is compared on the reference genome contains a deletion region of 100 bp in length. And for some reads containing large deletion regions do not display the deletion information directly in the cigar string when compared with the reference genome, they will be compared to two non-adjacent regions of the same chromosome by clipping the reads. Therefore, for the alignment with clipped reads, we need to set certain conditions to determine whether the alignment contains a deletion variant or not. If two segments of a read are aligned to the same chromosome in the same direction, the distance between the two segments on the read, Distance_read, is computed separately, and the distance between the two segments on the read, Distance_ref, is further computed for the two segments on the read aligned to the reference genome. Normally Distance_read should be 0, but due to sequencing errors and alignment tools, Distance_read may not be 0. In order to determine whether the alignment contains a deletion variant, it is necessary to determine whether the difference between them, Distance, is greater than 50; if Distance is greater than 50, then the alignment contains a deletion variant, but an upper threshold needs to be set for Distance. In order to explore a suitable upper threshold, we set different upper thresholds (Dt) to test the HG002 CLR dataset respectively, and the test results are shown in Table 8 below.

**TABLE 8:** LcDel results for different upper Distance thresholds on the CLR dataset.

Coverage		Dt ≤ 1,000	Dt ≤ 5,000	Dt ≤ 10,000	Dt ≤ 50,000	Dt ≤ 100,000	Dt ≤ 200,000
69X	precision	0.9627	0.9638	0.9642	0.9635	0.9611	0.9605
	recall	0.9239	0.9521	0.9761	0.9791	0.9832	0.9832
	F1	0.9429	0.9579	0.9701	0.9712	0.9721	0.9717
35X	precision	0.9495	0.9488	0.9495	0.9487	0.9485	0.9487
	recall	0.9195	0.9455	0.9696	0.9764	0.9764	0.9764
	F1	0.9343	0.9471	0.9594	0.9624	0.9623	0.9624
20X	precision	0.9381	0.9393	0.9403	0.9393	0.9369	0.9314
	recall	0.8877	0.9144	0.9378	0.9443	0.9446	0.9446
	F1	0.9122	0.9267	0.939	0.9406	0.9407	0.9379
10X	precision	0.9281	0.9287	0.9289	0.9286	0.9273	0.9286
	recall	0.8092	0.8386	0.8605	0.8663	0.8671	0.8671
	F1	0.8646	0.8813	0.8934	0.8961	0.8962	0.8964
5X	precision	0.9611	0.9614	0.9613	0.9612	0.9573	0.9505
	recall	0.637	0.6666	0.6819	0.6845	0.6866	0.6865
	F1	0.7662	0.7873	0.7978	0.7995	0.7997	0.7972

The above table shows that when the upper limit of the Distance threshold is set too small, LcDel has higher precision but lower recall in the detection results. As the upper limit of the Distance threshold continues to increase, the precision of the LcDel detection results gradually decreases, but the recall gradually increases. The reason for this analysis is that the upper limit of the Distance threshold is set too high causing many false positive events to be treated as missing events. If the upper Distance threshold is not set, LcDel will regard many false-positive events as deletion events, so it will ultimately lead to lower accuracy and higher recall of LcDel’s detection results. Therefore, setting an appropriate upper Distance threshold is especially important for the detection of missing variants, and the upper Distance threshold is set to 100,000 by the above table.

### 3.6 Comparison of deletion performance of LcDel at the first level of clustering using different methods

Since the length of large candidate deletion variant sites is generally longer, the effect may not be so obvious if the clustering is done using the coverage-based method, while the use of the sliding window-based clustering of deletion variants with larger lengths can have good results. Due to the short length of small candidate deletion sites, the deletion detection performance of LcDel may be reduced if a sliding window-based approach is directly used to cluster all deletion sites at the first level. In order to assess the impact of using the sliding window clustering method on small candidate deletion sites, we performed benchmarking on the first layer of clustering on the HG002 CLR dataset using sliding window based one method alone (SW) and using sliding window, coverage based two methods (SW + CG), the benchmarking results are shown in Table 9 below.

**TABLE 9:** Performance comparison of LcDel on different clustering methods.

Coverage		SW	SW + CG
69X	precision	0.9596	0.9611
	recall	0.9691	0.9832
	F1	0.9643	0.9721
35X	precision	0.9483	0.9485
	recall	0.9616	0.9764
	F1	0.9549	0.9623
20X	precision	0.9443	0.9369
	recall	0.9322	0.9446
	F1	0.9382	0.9407
10X	precision	0.8633	0.9273
	recall	0.8566	0.8671
	F1	0.86	0.8962
5X	precision	0.9062	0.9573
	recall	0.6766	0.6866
	F1	0.7747	0.7997

From Table 9, it can be seen that if all candidate deletion sites are clustered using the sliding window-based method, it does not have a better detection performance than clustering using both sliding window-based and coverage-based methods. Consequently, clustering of small candidate deletion sites using the sliding window-based method decreases the detection performance of LcDel, which may be due to the relatively large window setting, resulting in some small candidate deletion sites that are relatively close to each other being clustered together, affecting the accuracy of LcDel.

### 3.7 Comparison of detection performance of LcDel on HG002 CLR dataset for different window sizes

The use of sliding window based method in clustering large candidate deletion sites will achieve good results, but the setting of the window size may affect the detection performance of LcDel on deletion variants to a certain extent, so this paper attempted to set the sliding window (ws) to 500, 1,500, 2,500, 4,000 and 5,000 on the CLR dataset of HG002 for benchmarking, and the results are as follows in Table 10.

**TABLE 10:** Comparison of LcDel’s detection performance on different window sizes.

Coverage		ws = 500	ws = 1,500	ws = 2,500	ws = 4,000	ws = 5,000
69X	precision	0.9512	0.9611	0.9541	0.9635	0.9635
	recall	0.9627	0.9832	0.9811	0.9832	0.9832
	F1	0.9569	0.9721	0.9674	0.9733	0.9733
35X	precision	0.941	0.9485	0.9435	0.9485	0.9483
	recall	0.9657	0.9764	0.9744	0.9764	0.9764
	F1	0.9531	0.9623	0.9587	0.9622	0.9621
20X	precision	0.9315	0.9369	0.9393	0.9393	0.9393
	recall	0.9343	0.9446	0.9444	0.9443	0.9443
	F1	0.9328	0.9407	0.9418	0.9418	0.9418
10X	precision	0.9181	0.9273	0.9286	0.9286	0.9286
	recall	0.8556	0.8671	0.8663	0.8663	0.8663
	F1	0.8857	0.8962	0.8964	0.8961	0.8964
5X	precision	0.9518	0.9573	0.9608	0.9608	0.9601
	recall	0.6761	0.6866	0.6765	0.6763	0.6765
	F1	0.7906	0.7997	0.7937	0.7938	0.7937

Through Table 10, it can be found that when the window setting is small it will reduce the detection performance of LcDel, and when the window size is 1,500, no matter how to increase the window, it has little effect on the detection effect of LcDel, which is largely due to the hierarchical clustering in the second layer. If the setting of the window is very large, it will cluster many close deletion variants together to form a cluster, if the exact deletion site is determined directly at this time it will lead to misidentification or miss identification of the deletion variants, but the hierarchical clustering can separate them very well, so the effect of LcDel does not decrease with the increase of the window. Through analysis, LcDel sets the window size to 1,500.

## 4 Discussion

In this paper, we propose a long read based deletion variant detection method LcDel using two-layer clustering. LcDel first finds candidate deletion sites from the sorted bam file by intra-read alignment and inter-read alignment. A method of heuristics was used to merge relatively close deletion sites. Use sliding window and coverage methods based on deletion length to perform the first layer clustering and generate multiple large clusters. Then, hierarchical clustering is used to further cluster the large clusters and generate candidate clusters, in order to improve the accuracy of clustering and facilitate the determination of deletion positions and lengths in the future. Finally, the candidate clusters containing candidate deletion sites are filtered out, and the position and length of the deletion are determined from the remaining candidate clusters. To evaluate the detection performance of LcDel for deletion variants, we compared it with four currently popular structural variant detection tools on multiple datasets. The experimental results show that LcDel has better detection performance for deletions.

However, LcDel still has some limitations in some aspects. First, LcDel only detects deletion variants but not other types of structural variants such as insertions, translocations and inversions. Second, LcDel does not genotype the detected deletions. We will gradually improve the above problems in our future work.

## Data Availability

The original contributions presented in the study are included in the article/Supplementary material, further inquiries can be directed to the corresponding author.
